# Antipsychotic Treatment Among Youths With Attention-Deficit/Hyperactivity Disorder

**DOI:** 10.1001/jamanetworkopen.2019.7850

**Published:** 2019-07-26

**Authors:** Ryan S. Sultan, Shuai Wang, Stephen Crystal, Mark Olfson

**Affiliations:** 1 Department of Psychiatry, College of Physicians and Surgeons, Columbia University, New York, New York; 2 New York State Psychiatric Institute, New York; 3 Institute for Health, Health Care Policy, and Aging Research, Rutgers University, New Brunswick, New Jersey

## Abstract

**Importance:**

Significant concern exists over treating youths with attention-deficit/hyperactivity disorder (ADHD) with antipsychotic medications, yet little is known about the factors associated with antipsychotic treatment.

**Objectives:**

To describe the percentage of youths who fill antipsychotic prescriptions in the year following a new diagnosis of ADHD and characterize the clinical and demographic factors associated with antipsychotic initiation.

**Design, Setting, and Participants:**

A retrospective longitudinal cohort analysis of antipsychotic treatment was performed in 187 563 youths, aged 3 to 24 years, with a new diagnosis of ADHD (without recent diagnosis of any US Food and Drug Administration [FDA]–indicated conditions for antipsychotic treatment). The sample was derived from the 2010 to 2015 MarketScan Commercial Database, with the analysis completed between November 1, 2018, and May 30, 2019.

**Main Outcomes and Measures:**

The percentage of youths prescribed an antipsychotic in the first year following a new diagnosis of ADHD. Among those prescribed antipsychotic medications, the percentage who received a diagnosis of conduct disorder, oppositional defiant disorder, or a disorder for which 1 or more antipsychotic medication has received an indication for use in youths from the FDA (schizophrenia, bipolar disorder, and Tourette disorder) and the percentage that filled an antipsychotic prescription before filling a stimulant prescription (methylphenidate or amphetamine derivative).

**Results:**

Of the 187 563 youths included in the study, 114 305 (60.9%) were male with a mean (SD) age of 13.74 (5.61) years. In the year following a new ADHD diagnosis, 4869 youths (2.6%; 95% CI, 2.5%-2.7%) with ADHD were prescribed an antipsychotic. Youths treated with antipsychotics with ADHD were more likely than their peers who were not receiving an antipsychotic to have recently received diagnoses of self-harm and/or suicidal ideation (adjusted odds ratio [aOR], 7.5; 95% CI, 5.9-9.6), oppositional defiant disorder (aOR, 4.4; 95% CI, 3.9-4.9), and substance use disorder (aOR, 4.0; 95% CI, 3.6-4.5). The youths who received antipsychotics were also more likely to have received inpatient treatment (aOR, 7.9; 95% CI, 6.7-9.3). During the year following the new ADHD diagnosis, 52.7% (95% CI, 51.3%-54.1%) of youths treated with antipsychotics received a diagnosis for which antipsychotics have either an FDA or evidence-supported indication for their use. Among youths who initiated antipsychotic medications, 47.9% (95% CI, 46.5%-49.3%) did not receive a stimulant prescription between their ADHD diagnosis and antipsychotic initiation. Antipsychotic prescribing was proportionally highest for preschool-aged children (4.3%) and associated with neurodevelopmental disorders (aOR, 3.9; 95% CI, 1.3-11.2) and recent inpatient mental health treatment (aOR, 8.9; 95% CI, 1.7-45.8).

**Conclusions and Relevance:**

Approximately half of youths with a new ADHD diagnosis may have an evidence-supported indication for an antipsychotic medication. Less than half of these youths received a stimulant; the evidence-supported first line treatment for ADHD, before the antipsychotic was initiated. Use of antipsychotic prescribing appears to be associated with high levels of psychiatric comorbidity.

## Key Points

**Question:**

How commonly are youths with attention-deficit/hyperactivity disorder treated with antipsychotic medications and what factors are associated with their use of antipsychotic treatment?

**Findings:**

In this cohort study of 187 563 commercially insured youths with new episodes of attention-deficit/hyperactivity disorder, 2.3% were treated with an antipsychotic medication, among whom 52.7% had a potential clinical diagnostic rationale for antipsychotic treatment. Factors for antipsychotic medication use included older patient age, male sex, recent inpatient and other pharmacologic mental health treatments, self-harm/suicidal ideation; and oppositional defiant, substance use, depressive, and anxiety disorders.

**Meaning:**

Approximately 1 in 40 commercially insured youths were treated with an antipsychotic medication without an approved indication in the year following a new attention-deficit/hyperactivity disorder diagnosis; mental health comorbidities may be associated with antipsychotic treatment in this group.

## Teaser

This cohort study examines the use of antipsychotic medication in youths, aged 3 to 24 years, following a new diagnosis of attention-deficit/hyperactivity disorder.

## Introduction

In the past decade, there has been an increase in the prescription of antipsychotic medications to children and adolescents.^1,2,3,4^ Between 1999 and 2014, antipsychotic prescribing for young people in the United States increased by 50%.^4^ Some of the increase in antipsychotic medication use in youths was for clinical indications that have been approved by the US Food and Drug Administration (FDA), including schizophrenia, bipolar disorder, irritability associated with autistic disorder, and Tourette disorder.^5,6,7,8,9^ However, much of the increase has been associated with non–FDA-indicated prescribing, most commonly, attention-deficit/hyperactivity disorder (ADHD).^2,3,10,11^

Antipsychotic medications can have significant adverse effects in youths. With prolonged exposure, these medications frequently cause adverse metabolic effects, including weight gain, hyperlipidemia, and increased risk of type 2 diabetes.^2,12,13^ Recent evidence further suggests that antipsychotic treatment in youths is associated with an increased risk of unexpected death.^14^

Public and professional concern over the safety of antipsychotic use in young people has led to policies aimed at reducing use of these medications. Policy interventions have targeted absolute reductions in antipsychotic prescribing with less consideration of the clinical characteristics of patients treated with antipsychotics. As of 2014, most states had implemented prior authorization policies for use of antipsychotics in children in their Medicaid programs,^15,16^ with varying age limitations. These policies have lowered overall antipsychotic use in young children,^17^ although little is known about the clinical characteristics of young patients who are treated with antipsychotics in community practice.

Nearly half of all non–FDA-indicated antipsychotic medication use in young people is for individuals with ADHD diagnoses, with or without other mental health diagnoses.^2,18^ Yet, ADHD alone is not an evidence-supported indication for antipsychotic medications. Furthermore, little is known about which youths with ADHD are most likely to be treated with antipsychotic medications. Mental health comorbidity is one potential factor associated with antipsychotic treatment in youths with ADHD. Among Medicaid beneficiaries, youths treated with antipsychotics are often diagnosed with depression, oppositional defiant disorder (ODD), or conduct disorder (CD).^19^ Although depression is not an evidence-supported indication for antipsychotic treatment in youths, some evidence supports the effectiveness of risperidone for treatment of ODD or CD in stimulant-resistant youths with ADHD.^19,20,21,22,23,24,25,26,27^

The prevalence of antipsychotic use in youths following a new ADHD diagnosis is not known. Questions also exist regarding the extent to which comorbid mental disorders are associated with antipsychotic treatment in youths with ADHD. In addition, the frequency with which young people with ADHD receive antipsychotic medications before a trial of stimulants is not known. Closing these knowledge gaps will help to focus quality-of-care efforts aimed at safe and judicious antipsychotic prescribing.

We analyzed claims records of commercially insured youths, aged 3 to 24 years, with a new diagnosis of ADHD. Patients recently diagnosed with conditions for which antipsychotics have an FDA-approved indication (schizophrenia, bipolar disorder, autism spectrum disorder, and Tourette disorder) were excluded from the cohort. To better understand antipsychotic prescribing in this population, this study (1) determined the prevalence of antipsychotic use over the following year, (2) evaluated the associations between clinical and demographic factors and antipsychotic prescribing, and (3) examined the frequency with which stimulant treatment preceded antipsychotic treatment in the first year following a new ADHD diagnosis. Before conducting the analyses, we hypothesized that males and older youths would have higher rates of antipsychotic treatment. We further anticipated that most youths with ADHD who initiated antipsychotic medications would be diagnosed with comorbid, evidence-supported clinical diagnoses (ie, ODD or CD), and would fill stimulant prescriptions before initiating antipsychotic medications.

## Methods

### Data Source

Data were obtained from the Truven Health MarketScan database of commercially insured individuals from January 1, 2010, to December 31, 2015. This database comprises medical and prescription drug data for more than 115 million enrollees, including demographic characteristics, outpatient and inpatient service codes, diagnostic codes, and detailed pharmacy files.^28^ The data were deidentified and the study was determined to be exempt from human subjects review by the Rutgers University Institutional Review Board. This study followed the Strengthening the Reporting of Observational Studies in Epidemiology (STROBE) reporting guideline for cohort studies.

### Study Design, Participants, and Cohort Assembly

We examined records for youths with a new ADHD diagnosis (*International Classification of Diseases, Ninth Revision* (code 314.xx), and *International Statistical Classification of Diseases, 10th Revision* (code F90.xx), aged 3 to 24 years, after at least 120 days without an ADHD diagnosis. This 4-month period was chosen because youths with ADHD often are not seen by clinicians during summer months.^29^ Youths were excluded if they had been prescribed an antipsychotic or stimulant or had received a diagnosis with an indication for an antipsychotic medication (schizophrenia, bipolar disorder, autistic spectrum disorder, and Tourette disorder) in the previous 6 months. This process excluded 4555 youths (2.4% of the total sample). In addition, we required 12 months of continuous health care enrollment following the initial ADHD diagnosis.

### Clinical and Demographic Characteristics

#### Background Characteristics

Youths were characterized by sex, age at ADHD diagnosis (3-5, 6-12, 13-18, 19-24 years), other mental health clinical diagnoses (depression, substance use, anxiety, self-harm and/or suicidal ideation, developmental disability, ODD, and CD) and recent mental health treatment, including inpatient care, being seen by a psychiatrist, and other psychopharmacologic treatments during the 180 days before the initial ADHD diagnosis (eTable in the Supplement).

#### Diagnoses

Bipolar disorder, schizophrenia, and psychotic/delusional disorders, autism spectrum disorder, tic disorders, and Tourette disorder were considered as FDA-indicated diagnoses for antipsychotic treatment (eTable in the Supplement). Given the evidence of efficacy for risperidone in the treatment of ODD and CD, we considered these disorders to be evidence-supported diagnoses.^20,21,22,23,24,25,26,27^

### Statistical Analysis

The analysis was conducted in 5 stages between November 1, 2018, and May 30, 2019. We first examined the background (pre-ADHD diagnosis), clinical, and demographic characteristics of youths who did and did not receive an antipsychotic medication during the 12 months following the initial ADHD diagnosis. We examined the incidence of antipsychotic treatment in the year following a new ADHD diagnosis. Unadjusted and age- and sex-adjusted logistic regressions evaluated the associations of each clinical and demographic characteristic with antipsychotic treatment during the 12-month follow-up period.

Second, to characterize stimulant treatment before antipsychotic initiation, we calculated the percentages of youths who received stimulant prescriptions before antipsychotic initiation among youths initiating antipsychotic medications overall and stratified by sex and age group. Stimulant treatment was defined as no stimulant, only 1 stimulant class (methylphenidate or amphetamine derivative), or both classes (methylphenidate and amphetamine derivative) during the period between new a ADHD diagnosis and antipsychotic initiation.

Third, we examined the first antipsychotic initiated for the cohort who received treatment with antipsychotics. We calculated percentages for each agent and mean (SD) number of antipsychotic prescriptions during the 180-day follow-up period.

Fourth, restricted to the antipsychotic-treated cohort, we examined percentages of youths who received an FDA-indicated diagnosis in the year following new ADHD diagnosis (post-ADHD diagnosis) and percentages of youths who received evidence-supported diagnoses in the 6 months before and 12 months after the new ADHD diagnosis. These results were then stratified by age group.

Fifth, we performed a subgroup analysis of preschool or very young children (aged 3-5 years) from the initial cohort, using the same variables and adding neurodevelopmental disorders (eTable in the Supplement). Sixth, to assess time to antipsychotic treatment after a new ADHD diagnosis, a Kaplan-Meier survival analysis was performed for each age group (eFigure in the Supplement). Findings were considered significant at 2-tailed unpaired *P* < .05. All analyses were completed with SAS, version 7.15 HF7 (SAS Institute Inc).

## Results

Of the 187 563 youths included in the study, 114 305 (60.9%) were male, with a mean (SD) age of 13.74 (5.61) years. Within 1 year of a new ADHD diagnosis, antipsychotic medications were initiated by 4869 of 187 563 youths (2.6%; 95% CI, 2.5%-2.7%). The prevalence of antipsychotic treatment was 4.3% (95% CI, 3.9%-4.7%) for very young children (3-5 years), 2.0% (95% CI, 1.9%-2.1%) for older children (6-12 years), 3.2% (95% CI, 3.0%-3.3%) for adolescents (13-18 years), and 2.4% (95% CI, 2.3%-2.6%) for young adults (19-24 years). Overall, antipsychotic initiators were more likely to be male (114 305 [60.9%]); adolescent (13-18 years); recently diagnosed with comorbid mental health conditions, with the most frequent being anxiety (5.8%; 95% CI, 5.5%-6.2%); and recent admission for inpatient mental health treatment (17.6%; 95% CI, 15.3%-20.0%). Antipsychotic initiation was associated with self-harm and/or suicidal ideation (adjusted odds ratio [aOR], 7.5; 95% CI, 5.9-9.6), ODD diagnosis (aOR, 4.4; 95% CI, 3.9-4.9), substance use disorder (aOR, 4.0; 95% CI, 3.6-4.5), and inpatient mental health care (aOR, 7.9; 95% CI, 6.7-9.3) in the preceding 6 months (Table 1 and eFigure in the Supplement).

**Table 1.** Antipsychotic Treatment of Youths in the Year Following a New ADHD Diagnosis^a^

Groups^b^	No. (%)	Antipsychotic Treatment, % (95% CI) (n = 4342)	aOR (95% CI)^c^
Total	187 563	2.6 (2.5-2.7)	NA
Age, y			
3-5	10 125 (54.0)	4.3 (3.9-4.7)	2.2 (1.9-2.4)
6-12	72 126 (38.5)	2.0 (1.9-2.1)	1.0
13-18	58 166 (31.0)	3.2 (3.0-3.3)	1.6 (1.5-1.7)
19-24	47 146 (25.1)	2.4 (2.3-2.6)	1.2 (1.1-1.3)
Sex			
Male	114 305 (60.9)	2.7 (2.6-2.8)	1.1 (1.0-1.1)
Female	73 258 (39.1)	2.5 (2.4-2.6)	1 [Reference]
Comorbid MH condition^d^			
Oppositional defiant disorder	3269 (1.7)	10.1 (9.1-11.1)	4.4 (3.9-4.9)
Conduct disorder	5113 (2.7)	6.5 (5.8-7.2)	2.7 (2.4-3.0)
Anxiety disorder	17 261 (9.2)	5.8 (5.5-6.2)	2.7 (2.5-2.9)
Self-harm and/or suicidal ideation	454 (0.2)	17.4 (13.9-20.9)	7.5 (5.9-9.6)
Depression disorders	12 573 (6.7)	8.0 (7.6-8.5)	4.0 (3.7-4.3)
Substance use disorder	3851 (2.1)	9.1 (8.2-10.0)	4.0 (3.6-4.5)
Developmental disability	210 (0.01)	8.6 (4.8-12.4)	3.6 (2.2-5.8)
MH conditions, No.			
0	152 625 (81.4)	1.7 (1.7-1.8)	1 [Reference]
1	28 174 (15.0)	5.3 (5.1-5.6)	3.2 (3.0-3.4)
2	5876 (3.1)	9.7 (8.9-10.4)	6.1 (5.5-6.7)
≥3	888 (0.5)	17.1 (14.6-19.6)	11.5 (9.6-13.8)
MH treatment			
Inpatient care	1016 (0.5)	17.6 (15.3-20.0)	7.9 (6.7-9.3)
Treated by psychiatrist	25 866 (13.8)	5.9 (5.6-6.2)	2.9 (2.8-3.1)
Antidepressants	19 296 (10.3)	7.4 (7.0-7.7)	4.1 (3.8-4.4)
Mood stabilizers	3391 (1.8)	9.6 (8.6-10.5)	4.2 (3.7-4.7)
Hypnotics	5408 (2.9)	7.0 (6.4-7.7)	3.2 (2.8-3.6)

Abbreviations: ADHD, attention-deficit/hyperactivity disorder; aOR, adjusted odds ratio; MH, mental health; NA, not applicable.

^a^ 2010-2015, MarketScan Data.

^b^ Characteristics present within 180 days before a new ADHD diagnosis.

^c^ The aOR is adjusted by age and sex.

^d^ Groups are not exclusive.

Among youths who initiated antipsychotic medications, 47.9% (95% CI, 46.5%-49.3%) did not receive a stimulant prescription between their ADHD diagnosis and antipsychotic initiation. Approximately half of the youths (43.8%; 95% CI, 42.4%- 45.1%) who were treated with antipsychotics were prescribed 1 stimulant and 8.4% (95% CI, 7.6%-9.1%) were prescribed methylphenidate or a methylphenidate derivative and an amphetamine derivative before antipsychotic initiation (Table 2).

**Table 2.** Antipsychotic Treatment in Youths With ADHD Who Received Stimulant Treatment Before Antipsychotic Initiation^a^^,^^b^

Group	No.	Stimulant, % (95% CI)^c^^,^^d^		
		None	1 Class	Both Classes
Total	4869	47.9 (46.5-49.3)	43.8 (42.4-45.1)	8.4 (7.6-9.1)
Sex				
Male	3032	47.8 (46.0-49.6)	43.5 (41.7-45.2)	8.7 (7.7-9.8)
Female	1837	48.1 (45.8-50.4)	44.2 (41.9-46.5)	7.7 (6.5-9.0)
Age, y				
3-5	432	38.4 (33.8-43.0)	44.9 (40.2-49.6)	16.7 (13.2-20.2)
6-12	1459	46.7 (44.1-49.2)	42.2 (39.6-44.7)	11.2 (9.6-12.8)
13-18	1832	52.7 (50.4-55.0)	41.0 (38.7-43.3)	6.3 (5.2-7.5)
19-24	1146	45.4 (42.5-48.3)	49.7 (46.8-52.6)	4.9 (3.6-6.1)

Abbreviation: ADHD, attention-deficit/hyperactivity disorder.

^a^ 2010-2015, MarketScan Data.

^b^ Antipsychotic prescription during the 365 days following a new diagnosis of ADHD.

^c^ Presence or absence of any psychostimulant (methylphenidate or amphetamine derivate) on or before the first antipsychotic prescription.

^d^ Classes of the medications were methylphenidate and amphetamine derivative.

Table 3 presents the percentage of initial antipsychotic agents prescribed and the mean (SD) number of prescriptions. Risperidone (37.8%; mean [SD], 4.0 [3.5]), aripiprazole (32.0%; 3.6 [2.9]), and quetiapine (20.7%; 3.3 [3.0]) were the most commonly prescribed agents.

**Table 3.** Initial Antipsychotic Prescriptions for Study Period^a^

Antipsychotic Medication^b^	Prescriptions (n=4869)	
	%	Mean (SD)
All antipsychotics	100	4.1 (3.5)
Risperidone	37.8	4.0 (3.5)
Haloperidol	0.5	2.4 (2.1)
Quetiapine	20.7	3.3 (3.0)
Aripiprazole	32.0	3.6 (2.9)
Olanzapine	4.0	2.5 (2.3)
All other antipsychotics	5.0	2.7 (2.7)

Abbreviations: ADHD, attention-deficit/hyperactivity disorder.

^a^ 2010-2015 MarketScan Data (youths with new diagnosis of ADHD and antipsychotic prescription).

^b^ First antipsychotic prescription after diagnosis.

By the end of the follow-up year, 2565 of 19 990 youths (52.7%; 95% CI, 51.3%-54.1%) treated with antipsychotics had received either a diagnosis for which antipsychotics are FDA indicated in youths (35.1%; 95% CI, 33.8%-36.5%) or had received a clinical diagnosis for which there is evidence of antipsychotic efficacy in youths (26.9%; 95% CI, 25.6%-28.1%) (Table 4).

**Table 4.** Selected Clinical Diagnoses of Youths Treated With Antipsychotics With ADHD^a^

Selected Clinical Diagnosis	No. of Patients With ADHD	Youths Treated with Antipsychotics With ADHD, % (95% CI)					
		Total (n = 4869)	Age, y			
			3-5 (n = 432)	6-12 (n = 1459)	13-18 (n = 1832)	19-24 (n = 1146)
Any FDA-indicated or evidence-supported diagnosis	19 990	52.7 (51.3-54.1)	63.0 (58.4-67.5)	58.5 (56.0-61.1)	51.5 (49.2-53.8)	43.3 (40.4-46.2)
Any FDA-indicated diagnosis^b^		35.1 (33.8-36.5)	25.9 (21.8-30.1)	30.4 (28.1-32.8)	37.3 (35.1-39.6)	41.1 (38.3-44.0)
Bipolar disorder	2428	21.2 (20.0-22.3)	6.3 (4.0-8.5)	10.5 (8.9-12.1)	26.5 (24.5-28.5)	31.9 (29.2-34.6)
Psychosis	955	9.2 (8.4-10.0)	0.9 (0-1.8)	4.6 (3.5-5.7)	11.6 (10.2-13.1)	14.3 (12.3-16.3)
Autism spectrum disorder	3590	8.9 (8.1-9.7)	19.0 (15.3-22.7)	15.7 (13.8-17.6)	5.2 (4.2-6.3)	2.3 (1.4-3.1)
Tourette disorder	1568	2.2 (1.8-2.6)	2.1 (0.7-3.4)	3.6 (2.6-4.5)	1.8 (1.2-2.4)	1.0 (0.4-1.5)
Any evidence-supported diagnosis^b^^,^^c^		26.9 (25.6-28.1)	50.7 (46.0-55.4)	40.0 (37.5-42.5)	24.4 (22.4-26.4)	5.2 (3.9-6.4)
Oppositional defiant disorder	6374	16.1 (15.1-17.2)	28.7 (24.4-33.0)	25.9 (23.7-28.2)	15.0 (13.4-16.7)	0.8 (0.3-1.3)
Conduct disorder	8414	16.1 (15.1-17.1)	33.6 (29.1-38.0)	22.0 (19.9-24.1)	14.6 (13.0-16.2)	4.5 (3.3-5.6)

Abbreviations: ADHD, attention-deficit/hyperactivity disorder; FDA, US Food and Drug Administration.

^a^ 2010-2015 MarketScan Data.

^b^ Groups are not exclusive.

^c^ Includes pre-ADHD diagnosis study period and study year.

A total of 432 of 10 125 (4.3%) very young children (aged 3-5 years) with ADHD were treated with an antipsychotic medication. Compared with those who were not treated with an antipsychotic, those who received an antipsychotic were more likely to have been diagnosed with a neurodevelopment disorder (aOR, 3.9; 95% CI, 1.3-11.2), received inpatient mental health care (aOR, 8.9; 95% CI, 1.7-45.8), and received care from a psychiatrist (aOR, 3.3; 95% CI, 2.7-4.2) (Table 5). The likelihood of antipsychotic treatment in the very young cohort increased with the number of mental health and neurodevelopmental diagnoses they received (1 diagnosis: aOR, 1.9; 95% CI, 1.5-2.4; 2 diagnoses: aOR, 3.7; 95% CI, 2.4-5.7; ≥3 diagnoses: aOR, 6.8; 95% CI, 1.9-24.1).

**Table 5.** Very Young Children (3-5 Years) Prescribed Antipsychotics in the Year Following ADHD Diagnosis (n = 10 125)^a^

Characteristic^b^	Antipsychotic Treatment, No./Total No. (%)	aOR (95% CI)^c^
Total	432/10 125 (4.3)	NA
Sex		
Male	341/7666 (4.5)	1.2 (1.0-1.5)
Female	91/2459 (3.7)	1 [Reference]
Comorbid MH and neurodevelopmental conditions^d^		
None	223/6699 (3.3)	0.5 (0.4-0.6)
Oppositional defiant disorder	45/441 (10.2)	2.7 (2.0-3.8)
Conduct disorder	75/1040 (7.2)	1.9 (1.5-2.4)
Anxiety disorder (any)	33/413 (8.0)	2.1 (1.4-3.0)
Depression disorders (any)	5/56 (8.9)	2.2 (0.9-5.6)
Neurodevelopmental disorder	4/28 (14.3)	3.9 (1.3-11.2)
Developmental disability	1/21 (4.8)	1.1 (0.2-8.5)
No. of MH or neurodevelopmental conditions		
0	300/8358 (3.6)	1 [Reference]
1	105/1551 (6.8)	1.9 (1.5-2.4)
2	24/201 (11.9)	3.7 (2.4-5.7)
≥3	3/15 (20.0)	6.8 (1.9-24.1)
Treatment		
MH inpatient care	2/7 (28.6)	8.9 (1.7-45.8)
Treated by psychiatrist	116/1076 (10.8)	3.3 (2.7-4.2)
Treated by neurologist	11/353 (3.1)	0.7 (0.4-1.3)

Abbreviatons: ADHD, attention/deficit hyperactivity disorder; aOR, adjusted odds ratio; MH, mental health; NA, not applicable.

^a^ 2010-2015 MarketScan Data.

^b^ Characteristics present within 180 days before a new ADHD diagnosis.

^c^ The aOR is adjusted for sex.

^d^ Groups are not mutually exclusive.

The eFigure in the Supplement displays the time to antipsychotic initiation for ADHD youths by age group, with the most rapid increase occurring during the first 30 days.

## Discussion

Antipsychotic medications were prescribed for 2.6% of commercially insured youths in the year following a new clinical diagnosis of ADHD without a recent diagnosis that has an FDA indication for antipsychotic treatment. Factors for antipsychotic treatment included a wide range of comorbid psychiatric diagnoses. Conduct disorder and ODD were factors associated with antipsychotic treatment. However, only approximately half of the antipsychotic treated patients received diagnoses (FDA-indicated, CD or ODD) with evidence of clinical benefits from antipsychotics during the pre- or post-ADHD diagnosis period. These data reveal the extent to which antipsychotics are prescribed to young people with ADHD and raise concerns regarding potential non–evidence-based antipsychotic use and underuse of stimulant medications.

### Prevalence of Antipsychotic Use in ADHD

The percentage of youths with a new ADHD diagnosis who were treated with an antipsychotic without an FDA indication (2.6%) was greater than the overall annual prevalence of antipsychotic treatment among youths in the general population (0.6%).^4,30,31^ Despite public policies and interventions aimed at reducing off-label antipsychotic medication prescriptions for youths,^32,33^ young people diagnosed with ADHD appear to have an increased likelihood of filling antipsychotic prescriptions. Professional statements and practice guidelines released by the American Psychiatric Association and the American Academy of Child and Adolescent Psychiatry have encouraged clinicians to reduce use of antipsychotic medications in youths without an approved or evidence-supported indication.^23,34^ Although these efforts may have reduced off-label prescription of antipsychotic medications to young people overall, their use among young people with ADHD remains significant.

### Age-Related Changes in Antipsychotic Use

The demographic profile of antipsychotic prescribing among youths with ADHD diagnoses is consistent with patterns observed in the general population of young people. Within the general population, the likelihood of antipsychotic use is also higher among male than female individuals and adolescents than children.^2^ This pattern may be associated with the use of antipsychotics in the management of impulsive and aggressive behaviors in youths with ADHD.^18,20^ After adolescence, decreases in the fraction of antipsychotic prescribing for young adults with ADHD have previously been described and are consistent with treatment of conduct problems that peak in adolescence.^35^ This decline is potentially associated with suppression of aggressive behaviors and improved impulse control in late adolescence from maturation of prefrontal gray matter and proliferation of white matter tracts.^36,37^

The flattest antipsychotic initiation curve occurs in school-aged youths (6-12 years). Based on new incidence, this is the age group at highest risk for initial ADHD presentations and when most medications are first used.^18^ A possible explanation of a flat curve is that symptoms are being addressed through the availability of nonpharmacologic, school-based interventions available to elementary school children.^38^ However, because these interventions are not recorded through insurance databases, we are unable to measure this.

### Diagnostic Factors Associated With Antipsychotic Use

Among youths with ADHD, several clinically diagnosed mental disorder comorbidities have been associated with an elevated likelihood for antipsychotic treatment. In youths with ADHD, many of these comorbidities are related to poorer response to stimulant treatment and adverse outcomes.^39,40,41^ Although antipsychotics could be prescribed because of incomplete response to other treatments, many of these conditions do not have empirical evidence supporting antipsychotic treatment. Outside of FDA indications, only ODD, CD, and aggression in ADHD have clinical experimental evidence to support the use of antipsychotics and only approximately half of the cohort of youths with ADHD who received antipsychotics had 1 or more of these clinical diagnoses.^20,21,22,23,24,25,26,27^

Recent inpatient mental health care was associated with antipsychotic treatment. Symptom severity thresholds for receiving acute inpatient mental health care are often high, particularly for severe ADHD.^42,43,44^ Young people whose symptoms have led to hospitalization likely have more complex clinical presentations than their peers who are not receiving antipsychotics. However, the effectiveness of antipsychotics for the treatment of severe uncomplicated ADHD has not been established. The youths with ADHD who were prescribed an antipsychotic but lacked an ODD or CD diagnosis could also have aggression or represent poor community pharmacologic management of ADHD.

Antipsychotic treatment was proportionately highest among very young children (4.3%). This group accounts for approximately a quarter of a percent of the total ADHD cohort, with very young children accounting for a small percentage of ADHD-treated youths. These children were more likely to have neurodevelopmental disorders than their age-matched peers who were not treated with antipsychotic medications and received high rates of inpatient mental health care. Antipsychotic treatment may be reserved for children with greater symptom severity. While ADHD uncommonly presents in very young children, those with ADHD tend to have severe symptoms and relatively poor response rates to stimulants.^45,46^ Furthermore, although nonpharmacologic treatment is generally regarded as first line in this age group, evidence-based psychosocial interventions, such as parenting training and parent-child interaction therapy, are not widely available.^47^

Fewer than half of the youths received a stimulant before their antipsychotic and fewer than 1 in 10 received a methylphenidate and amphetamine derivative. Antipsychotics are only potentially indicated in youths with ADHD after treatment trials of both methylphenidate and mixed amphetamine salts.^21,48^ This treatment pattern raises concerns that some youths with ADHD may be treated with antipsychotics prematurely, before receiving trials of adequate dose and duration of both classes of stimulants, potentially unnecessarily exposing them to antipsychotic treatment.

Risperidone was the most commonly first prescribed antipsychotic for youths with ADHD. This practice is consistent with recommendations. Risperidone has the strongest evidence base for off-label use in youths, particularly in ODD, CD, and aggression.^21,22,23,24,25,26,27^ However, nearly two-thirds of the antipsychotic-treated sample initially were treated with other antipsychotics that either have negative trials or lack trials to support their use in ADHD.^25,26,27^

A wide range of clinical characteristics, including mental disorder comorbidities, and inpatient mental health care are associated with antipsychotic treatment in youths with ADHD. Aggression, which is common in ADHD and not always responsive to stimulant medications, has been hypothesized to account for much of the off-label use of antipsychotics in youths with ADHD.^2,49,50^ Consistent with this hypothesis, antipsychotic-treated youths with ADHD had clinical characteristics commonly associated with aggression in ADHD, such as inpatient hospitalization and a higher prevalence of comorbid disorders.^42,43,44,51,52,53^ However, few youths treated with antipsychotics with ADHD received the evidence-indicated trials of 2 stimulants before an antipsychotic.

Current therapy management efforts in both private and publicly insured populations have been focused on curbing antipsychotic use in youths with less attention devoted to the clinical rationale for use. These policies have been particularly widely applied to Medicaid populations,^15^ including telephone consultations with psychiatrists, letters to prescribers, and age-related prior authorization policies.^15^ A greater understanding of the clinical rationale and timing of antipsychotic treatment of youths with ADHD might help to guide more clinically sensitive policies to regulate antipsychotic use.

This analysis was limited to commercially insured children. Much of the prior work on antipsychotic use in children has focused on Medicaid populations. Important socioeconomic and health differences exist between commercially insured and Medicaid-insured populations.^54,55^ Yet some aspects of our sample resemble those in antipsychotic-treated Medicaid youths. For example, similar to analyses of Medicaid samples, our sample was predominantly male and had similar rates of CD, anxiety, and depression diagnoses.^14,19,56,57^

### Limitations

This analysis has several limitations. First, diagnoses were based on clinical judgment—not structured interviews. Second, we were unable to examine antipsychotic treatment following the first lifetime diagnosis of ADHD. Patterns of antipsychotic treatment may be different following initial and new episodes of clinical ADHD diagnosis. Third, we examined commercially insured individuals; different results might be obtained from Medicaid beneficiaries in whom antipsychotic treatment is more common.^57^ Although Medicaid programs have implemented management programs to limit antipsychotic use, few commercial insurance programs have followed suit. Nevertheless, these programs, along with medical society guidelines,^48^ may be associated with diagnostic and prescribing practices for commercially insured patients. Fourth, several potentially important factors to understanding antipsychotic treatment patterns, such as race/ethnicity, family circumstances, and home environment, were unavailable in the data set. Furthermore, we were unable to measure symptoms that might have provided a clinical rationale, such as severe aggression, for antipsychotic treatment. In addition, data on medication use were based on filled prescriptions rather than observed medication consumption.

## Conclusions

Among diagnostic groups in young people, ADHD may account for the largest share of antipsychotic treatment.^2,3,10,11^ Our results suggest associations between comorbid psychiatric diagnoses and the use of antipsychotic treatment in youths with ADHD. While some of these young people were diagnosed with conditions for which antipsychotics have some evidence of clinical effectiveness, many others were not. In view of the serious safety risks posed by antipsychotic treatment of young people, these patterns highlight the importance of remaining vigilant concerning antipsychotic medication prescribing patterns in the community treatment of ADHD.

## Notes

Supplement. **eTable.** Coding For MarketScan Data (2010-2015) **eFigure.** Survival Curve Comparing Age Group to Time of Antipsychotic Initiation in Youths With a New ADHD Diagnosis

## References

[1] Olfson M, Blanco C, Wang S, Laje G, Correll CU National trends in the mental health care of children, adolescents, and adults by office-based physicians. JAMA Psychiatry. 2014;71(1):-. doi:10.1001/jamapsychiatry.2013.3074 24285382

[2] Olfson M, King M, Schoenbaum M Treatment of young people with antipsychotic medications in the United States. JAMA Psychiatry. 2015;72(9):867-874. doi:10.1001/jamapsychiatry.2015.0500 26132724

[3] Olfson M, Blanco C, Liu SM, Wang S, Correll CU National trends in the office-based treatment of children, adolescents, and adults with antipsychotics. Arch Gen Psychiatry. 2012;69(12):1247-1256. doi:10.1001/archgenpsychiatry.2012.647 22868273

[4] Hales CM, Kit BK, Gu Q, Ogden CL Trends in prescription medication use among children and adolescents—United States, 1999-2014. JAMA. 2018;319(19):2009-2020. doi:10.1001/jama.2018.5690 29800213 PMC6583241

[5] Marcus RN, Owen R, Kamen L, A placebo-controlled, fixed-dose study of aripiprazole in children and adolescents with irritability associated with autistic disorder. J Am Acad Child Adolesc Psychiatry. 2009;48(11):1110-1119. doi:10.1097/CHI.0b013e3181b76658 19797985

[6] McCracken JT, McGough J, Shah B, ; Research Units on Pediatric Psychopharmacology Autism Network Risperidone in children with autism and serious behavioral problems. N Engl J Med. 2002;347(5):314-321. doi:10.1056/NEJMoa013171 12151468

[7] Findling RL, Robb A, Nyilas M, A multiple-center, randomized, double-blind, placebo-controlled study of oral aripiprazole for treatment of adolescents with schizophrenia. Am J Psychiatry. 2008;165(11):1432-1441. doi:10.1176/appi.ajp.2008.07061035 18765484

[8] Tohen M, Kryzhanovskaya L, Carlson G, Olanzapine versus placebo in the treatment of adolescents with bipolar mania. Am J Psychiatry. 2007;164(10):1547-1556. doi:10.1176/appi.ajp.2007.06111932 17898346

[9] Findling RL, Nyilas M, Forbes RA, Acute treatment of pediatric bipolar I disorder, manic or mixed episode, with aripiprazole: a randomized, double-blind, placebo-controlled study. J Clin Psychiatry. 2009;70(10):1441-1451. doi:10.4088/JCP.09m05164yel 19906348

[10] Alexander GC, Gallagher SA, Mascola A, Moloney RM, Stafford RS Increasing off-label use of antipsychotic medications in the United States, 1995-2008. Pharmacoepidemiol Drug Saf. 2011;20(2):177-184. doi:10.1002/pds.2082 21254289 PMC3069498

[12] Matone M, Localio R, Huang Y-S, dosReis S, Feudtner C, Rubin D The relationship between mental health diagnosis and treatment with second-generation antipsychotics over time: a national study of US Medicaid-enrolled children. Health Serv Res. 2012;47(5):1836-1860. doi:10.1111/j.1475-6773.2012.01461.x 22946905 PMC3513608

[13] Correll CU, Lencz T, Malhotra AK Antipsychotic drugs and obesity. Trends Mol Med. 2011;17(2):97-107. doi:10.1016/j.molmed.2010.10.010 21185230 PMC3053585

[14] Galling B, Correll CU Do antipsychotics increase diabetes risk in children and adolescents? Expert Opin Drug Saf. 2015;14(2):219-241. doi:10.1517/14740338.2015.979150 25480466

[15] Ray WA, Stein CM, Murray KT, Association of antipsychotic treatment with risk of unexpected death among children and youths. JAMA Psychiatry. JAMA Psychiatry. 2019;76(2):162-171. doi:10.1001/jamapsychiatry.2018.3421 30540347 PMC6440238

[16] Schmid I, Burcu M, Zito JM Medicaid prior authorization policies for pediatric use of antipsychotic medications. JAMA. 2015;313(9):966-968. doi:10.1001/jama.2015.0763 25734740

[17] Melvin KE, Hart JC, Sorvig RD Second-generation antipsychotic prescribing patterns for pediatric patients enrolled in West Virginia Medicaid. Psychiatr Serv. 2017;68(10):1061-1067. doi:10.1176/appi.ps.201600489 28566023

[18] Zito JM, Burcu M, McKean S, Warnock R, Kelman J Pediatric use of antipsychotic medications before and after Medicaid peer review implementation. JAMA Psychiatry. 2018;75(1):100-103. doi:10.1001/jamapsychiatry.2017.3493 29141077 PMC5833529

[19] Sultan RS, Correll CU, Schoenbaum M, King M, Walkup JT, Olfson M National patterns of commonly prescribed psychotropic medications to young people. J Child Adolesc Psychopharmacol. 2018;28(3):158-165. doi:10.1089/cap.2017.0077 29376743 PMC5905871

[20] Pathak P, West D, Martin BC, Helm ME, Henderson C Evidence-based use of second-generation antipsychotics in a state Medicaid pediatric population, 2001-2005. Psychiatr Serv. 2010;61(2):123-129. doi:10.1176/ps.2010.61.2.123 20123816

[21] Pan P-Y, Fu A-T, Yeh C-B Aripiprazole/methylphenidate combination in children and adolescents with disruptive mood dysregulation disorder and attention-deficit/hyperactivity disorder: an open-label study. J Child Adolesc Psychopharmacol. 2018;28(10):682-689. doi:10.1089/cap.2018.0068 30148656

[22] Gurnani T, Ivanov I, Newcorn JH Pharmacotherapy of aggression in child and adolescent psychiatric disorders. J Child Adolesc Psychopharmacol. 2016;26(1):65-73. doi:10.1089/cap.2015.0167 26881859

[23] Pringsheim T, Hirsch L, Gardner D, Gorman DA The pharmacological management of oppositional behaviour, conduct problems, and aggression in children and adolescents with attention-deficit hyperactivity disorder, oppositional defiant disorder, and conduct disorder: a systematic review and meta-analysis—part 2: antipsychotics and traditional mood stabilizers. Can J Psychiatry. 2015;60(2):52-61. doi:10.1177/070674371506000203 25886656 PMC4344947

[24] Findling RL, Drury SS, Jensen PS AACAP Committee on Quality Issues. Practice parameter for the use of atypical antipsychotic medications in children and adolescents. https://www.aacap.org/App_Themes/AACAP/docs/practice_parameters/Atypical_Antipsychotic_Medications_Web.pdf. Published 2011. Accessed June 18, 2019.

[25] Armenteros JL, Lewis JE, Davalos M, Arabgol F, Panaghi L, Nikzad V Risperidone augmentation for treatment-resistant aggression in attention-deficit/hyperactivity disorder: a placebo-controlled pilot study. J Am Acad Child Adolesc Psychiatry. 2007;46(5):558-565. doi:10.1097/chi.0b013e3180323354 17450046

[26] Findling RL, Short EJ, Leskovec T, Aripiprazole in children with attention-deficit/hyperactivity disorder. J Child Adolesc Psychopharmacol. 2008;18(4):347-354. doi:10.1089/cap.2007.0124 18759644

[27] Tramontina S, Zeni CP, Ketzer CR, Pheula GF, Narvaez J, Rohde LA Aripiprazole in children and adolescents with bipolar disorder comorbid with attention-deficit/hyperactivity disorder: a pilot randomized clinical trial. J Clin Psychiatry. 2009;70(5):756-764. doi:10.4088/JCP.08m04726 19389329

[28] Kronenberger WG, Giauque AL, Lafata DE, Bohnstedt BN, Maxey LE, Dunn DW Quetiapine addition in methylphenidate treatment-resistant adolescents with comorbid ADHD, conduct/oppositional-defiant disorder, and aggression: a prospective, open-label study. J Child Adolesc Psychopharmacol. 2007;17(3):334-347. doi:10.1089/cap.2006.0012 17630867

[29] Leigh Hansen MSM The Truven Health MarketScan Databases for life sciences researchers. https://truvenhealth.com/Portals/0/Assets/2017-MarketScan-Databases-Life-Sciences-Researchers-WP.pdf. Published March 2017. Accessed February 12, 2018.

[30] Ibrahim K, Donyai P Drug holidays from ADHD medication: international experience over the past four decades. J Atten Disord. 2015;19(7):551-568. doi:10.1177/1087054714548035 25253684

[31] Froehlich TE, Lanphear BP, Epstein JN, Barbaresi WJ, Katusic SK, Kahn RS Prevalence, recognition, and treatment of attention-deficit/hyperactivity disorder in a national sample of US children. Arch Pediatr Adolesc Med. 2007;161(9):857-864. doi:10.1001/archpedi.161.9.857 17768285

[32] Olfson M, Gameroff MJ, Marcus SC, Jensen PS National trends in the treatment of attention deficit hyperactivity disorder. Am J Psychiatry. 2003;160(6):1071-1077. doi:10.1176/appi.ajp.160.6.1071 12777264

[33] Vitiello B, Correll C, van Zwieten-Boot B, Zuddas A, Parellada M, Arango C Antipsychotics in children and adolescents: increasing use, evidence for efficacy and safety concerns. Eur Neuropsychopharmacol. 2009;19(9):629-635. doi:10.1016/j.euroneuro.2009.04.008 19467582

[34] Correll CU Antipsychotic use in children and adolescents: minimizing adverse effects to maximize outcomes. J Am Acad Child Adolesc Psychiatry. 2008;47(1):9-20. doi:10.1097/chi.0b013e31815b5cb1 18174821

[35] Choosing Wisely American Psychiatric Association. http://www.choosingwisely.org/clinician-lists/american-psychiatric-association-antipsychotics-in-children-or-adolescents/. Updated August 21, 2014. Accessed June 1, 2019. .

[36] Moffitt TE Adolescence-limited and life-course-persistent antisocial behavior: a developmental taxonomy.Psychol Rev. 1993;100(4):674-701. doi:10.1037/0033-295X.100.4.674 8255953

[37] Monahan KC, Steinberg L, Cauffman E, Mulvey EP Trajectories of antisocial behavior and psychosocial maturity from adolescence to young adulthood. Dev Psychol. 2009;45(6):1654-1668. doi:10.1037/a0015862 19899922 PMC2886970

[38] Steinberg L A social neuroscience perspective on adolescent risk-taking. Dev Rev. 2008;28(1):78-106. doi:10.1016/j.dr.2007.08.002 18509515 PMC2396566

[39] Slade EP Effects of school-based mental health programs on mental health service use by adolescents at school and in the community. Ment Health Serv Res. 2002;4(3):151-166. doi:10.1023/A:1019711113312 12385568

[40] Kessler RC, Adler LA, Berglund P, The effects of temporally secondary co-morbid mental disorders on the associations of *DSM-IV* ADHD with adverse outcomes in the US National Comorbidity Survey Replication Adolescent Supplement (NCS-A). Psychol Med. 2014;44(8):1779-1792. doi:10.1017/S0033291713002419 24103255 PMC4124915

[41] Blader JC, Pliszka SR, Jensen PS, Schooler NR, Kafantaris V Stimulant-responsive and stimulant-refractory aggressive behavior among children with ADHD. Pediatrics. 2010;126(4):e796-e806. doi:10.1542/peds.2010-0086 20837589 PMC2956067

[42] Al Ghriwati N, Langberg JM, Gardner W, Impact of mental health comorbidities on the community-based pediatric treatment and outcomes of children with attention deficit hyperactivity disorder. J Dev Behav Pediatr. 2017;38(1):20-28. doi:10.1097/DBP.0000000000000359 27902542 PMC5198773

[43] Souery D, Oswald P, Massat I, ; Group for the Study of Resistant Depression Clinical factors associated with treatment resistance in major depressive disorder: results from a European multicenter study. J Clin Psychiatry. 2007;68(7):1062-1070. doi:10.4088/JCP.v68n0713 17685743

[44] Martin A, Leslie D Psychiatric inpatient, outpatient, and medication utilization and costs among privately insured youths, 1997-2000. Am J Psychiatry. 2003;160(4):757-764. doi:10.1176/appi.ajp.160.4.757 12668366

[45] Tossone K, Jefferis E, Bhatta MP, Bilge-Johnson S, Seifert P. Risk factors for rehospitalization and inpatient care among pediatric psychiatric intake response center patients. Child Adolesc Psychiatry Ment Health. 2014;8(1):27. doi:10.1186/1753-2000-8-27 25392713 PMC4228274

[46] Greenhill L, Kollins S, Abikoff H, Efficacy and safety of immediate-release methylphenidate treatment for preschoolers with ADHD. J Am Acad Child Adolesc Psychiatry. 2006;45(11):1284-1293. doi:10.1097/01.chi.0000235077.32661.61 17023867

[47] Vitiello B, Lazzaretto D, Yershova K, Pharmacotherapy of the Preschool ADHD Treatment Study (PATS) children growing up. J Am Acad Child Adolesc Psychiatry. 2015;54(7):550-556. doi:10.1016/j.jaac.2015.04.004 26088659 PMC4475273

[48] Comer JS, Furr JM, Miguel EM, Remotely delivering real-time parent training to the home: an initial randomized trial of Internet-delivered parent-child interaction therapy (I-PCIT). J Consult Clin Psychol. 2017;85(9):909-917. doi:10.1037/ccp0000230 28650194

[49] Scotto Rosato N, Correll CU, Pappadopulos E, Chait A, Crystal S, Jensen PS; Treatment of Maladaptive Aggressive in Youth Steering Committee Treatment of maladaptive aggression in youth: CERT guidelines II—treatments and ongoing management. Pediatrics. 2012;129(6):e1577-e1586. doi:10.1542/peds.2010-1361 22641763

[50] Connor DF, Glatt SJ, Lopez ID, Jackson D, Melloni RH Jr Psychopharmacology and aggression—I: a meta-analysis of stimulant effects on overt/covert aggression-related behaviors in ADHD. J Am Acad Child Adolesc Psychiatry. 2002;41(3):253-261. doi:10.1097/00004583-200203000-00004 11886019

[51] Jensen PS, Youngstrom EA, Steiner H, Consensus report on impulsive aggression as a symptom across diagnostic categories in child psychiatry: implications for medication studies. J Am Acad Child Adolesc Psychiatry. 2007;46(3):309-322. doi:10.1097/chi.0b013e31802f1454 17314717

[52] Crystal S, Olfson M, Huang C, Pincus H, Gerhard T Broadened use of atypical antipsychotics: safety, effectiveness, and policy challenges. Health Aff (Millwood). 2009;28(5):w770-w781. doi:10.1377/hlthaff.28.5.w770 19622537 PMC2896705

[53] O’Leary KD, Tintle N, Bromet EJ, Gluzman SF Descriptive epidemiology of intimate partner aggression in Ukraine. Soc Psychiatry Psychiatr Epidemiol. 2008;43(8):619-626. doi:10.1007/s00127-008-0339-8 18360731

[54] Connor DF, Chartier KG, Preen EC, Kaplan RF Impulsive aggression in attention-deficit/hyperactivity disorder: symptom severity, co-morbidity, and attention-deficit/hyperactivity disorder subtype. J Child Adolesc Psychopharmacol. 2010;20(2):119-126. doi:10.1089/cap.2009.0076 20415607 PMC5695738

[55] Frakt A, Carroll AE, Pollack HA, Reinhardt U Our flawed but beneficial Medicaid program. N Engl J Med. 2011;364(16):e31. doi:10.1056/NEJMp1103168 21470001

[56] Todd J, Armon C, Griggs A, Poole S, Berman S Increased rates of morbidity, mortality, and charges for hospitalized children with public or no health insurance as compared with children with private insurance in Colorado and the United States. Pediatrics. 2006;118(2):577-585. doi:10.1542/peds.2006-0162 16882810

[57] Bobo WV, Cooper WO, Stein CM, Antipsychotics and the risk of type 2 diabetes mellitus in children and youth. JAMA Psychiatry. 2013;70(10):1067-1075. doi:10.1001/jamapsychiatry.2013.2053 23965896

[58] Crystal S, Mackie T, Fenton MC, Rapid growth of antipsychotic prescriptions for children who are publicly insured has ceased, but concerns remain. Health Aff (Millwood). 2016;35(6):974-982. doi:10.1377/hlthaff.2016.0064 27269012

