# Numeric Error in Key Points Findings

**DOI:** 10.1001/jamanetworkopen.2019.11650

**Published:** 2019-08-28

In the Original Investigation titled “Antipsychotic Treatment Among Youths With Attention-Deficit/Hyperactivity Disorder,”^1^ published on July 26, 2019, there was a numeric error in the Key Points Findings. The number given as “2.3%” should have been “2.6%.” This article has been corrected.^1^
