# Supplementary material for: Antipsychotic Treatment Among Youths With Attention-Deficit/Hyperactivity Disorder
Source: JAMA Netw Open. 2019 Jul 26;2(7):e197850. doi: 10.1001/jamanetworkopen.2019.7850 (PMC6661708; doi:10.1001/jamanetworkopen.2019.7850)
Supplement: Supplement. — eTable. Coding For MarketScan Data (2010-2015) eFigure. Survival Curve Comparing Age Group to Time of Antipsychotic Initiation in Youths With a New ADHD Diagnosis [file jamanetwopen-2-e197850-s001.pdf]

Supplementary Online Content

Sultan RS, Wang S, Crystal S, Olfson M. Antipsychotic treatment among youths with attention-deficit/hyperactivity disorder. *JAMA Netw Open*. 2019;2(7):e197850. doi:10.1001/jamanetworkopen.2019.7850

eTable. Coding For MarketScan Data (2010-2015)

eFigure. Survival Curve Comparing Age Group to Time of Antipsychotic Initiation in Youth With a New ADHD Diagnosis

This supplementary material has been provided by the authors to give readers additional information about their work.

© 2019 Sultan RS et al. *JAMA Network Open.*

eTable. Coding for MarketScan Data (2010-2015)

**Characteristics**	**MarketScan Coding**
Bipolar Disorder	296.0x, 296.1x, 296.4x, 296.5x, 296.6x, 296.7x, 296.8x, (F30-F31.xx)
Psychosis	295.xx, 297.X, 298.XX, (F20.xx- F29.xx)
Autism Spectrum Disorder	299.XX, (F84.xx)
Tourette's Disorder	307.2x (F95.xx)
Oppositional Defiant Disorder	313.81 (F91.3)
Conduct Disorder	312.XX (F91.x, excluding F91.3)
Anxiety Disorder	300.0x, 300.2x, 300.3, 309.21, 300.09, 300.20-300.23, 300.29, 300.3, 300.7, 312.39, 313.0, 313.21, 313.23 (F41.xx, F42.xx, F93.x, F40.x, F45.xx, F63.3, F94.0)
Self-harm / Suicidality	E950-E959, V62.84, (X71-X83, R45.851)
Depression Disorder	296.2x, 296.3x, 298.0, 300.4, 311 (F32.xx-F34.xx)
Substance use Disorder	303-305, 303.xx, 305, 304.xx (F10.xx – F19.xx)
Developmental Disability	317.xx – 319.xx (F70-F79)
Inpatient Mental Health Care	(STDPLAC 21 or 51) and Any inpatient discharge with a first listed mental disorder (290-319) (F01-F99)
Neuro Developmental Disorder	Prader Willi (759.81,Q87.1), Tuberous sclerosis (759.5, Q85.1), Fetal alcohol syndrome (760.71, Q86.0), Fragile X (759.83, Q99.2), Cerebral Palsy (343.8, G80.4), taxia-telangiectasia (334.8, G11.3), Neurofibromatosis (759.6, Q85.8), Metachromatic Leukodystrophy (330.0, E75.25) Wilson's Disease (275.1, E83.01), Huntington's Disease (333.4, G10) Krabbe's Disease (330.0, E75.23)

© 2019 Sultan RS et al. *JAMA Network Open.*

eFigure. Survival Curve Comparing Age Group to Time of Antipsychotic Initiation in Youth With a New ADHD Diagnosis

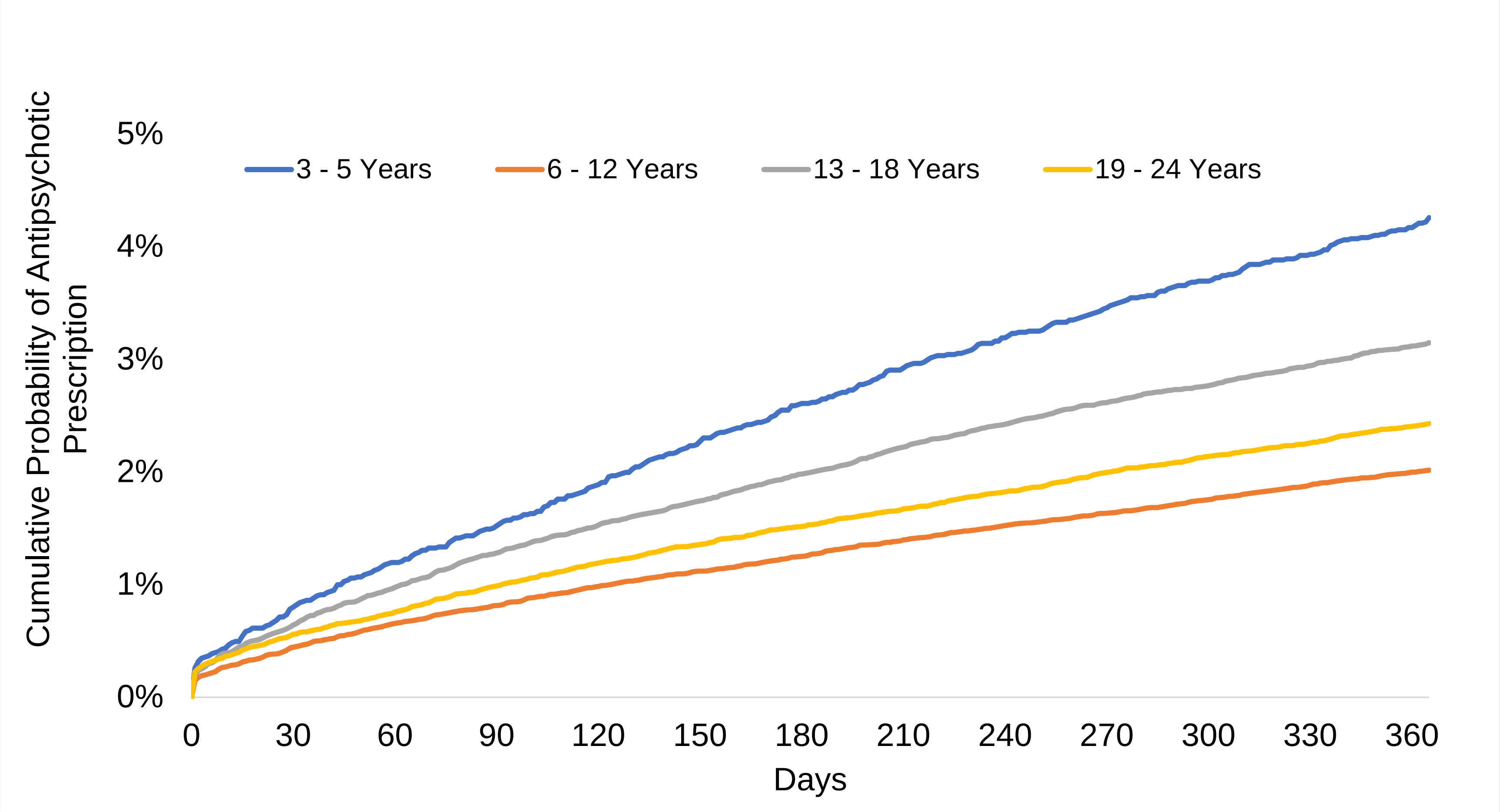

Source: 2010-2015, MarketScan Data. Sample Sizes: 3-5 Years (415), 6-12 Years (1,264), 13-18 Years (1,629), 19-24 Years (1,000). All groups were signficantly different from each other (P<0.001).

© 2019 Sultan RS et al. *JAMA Network Open.*
